# Human cardiac organoids: A recent revolution in disease modeling and regenerative medicine

**DOI:** 10.34172/jcvtr.2023.31830

**Published:** 2023-06-29

**Authors:** Neda Roshanravan, Samad Ghaffari, Sepideh Bastani, Sara Pahlavan, Samira Asghari, Mohammad Amin Doustvandi, Sepideh Jalilzadeh- Razin, Mohammadreza Dastouri

**Affiliations:** ^1^Cardiovascular Research Center, Tabriz University of Medical Sciences, Tabriz, Iran; ^2^Department of Immunology, Leiden University Medical Science, Leiden, Netherlands; ^3^Department of Stem Cells and Developmental Biology, Cell Science Research Center, Royan Institute for Stem Cell Biology and Technology, ACECR, Tehran, Iran; ^4^University of Social Welfare and Rehabilitation Sciences, Tehran, Iran; ^5^Immunology Research Center, Tabriz University of Medical Sciences, Tabriz, Iran; ^6^Research Center for Pharmaceutical Nanotechnology, Biomedicine Institute, Tabriz University of Medical Sciences, Tabriz, Iran; ^7^Ankara University Biotechnology Institute and SISBIYOTEK Advanced Research Unit, Gumusdere Yerleskesi, Kecioren, Ankara, Turkey

**Keywords:** Organoid, Regenerative Medicine, Three-Dimensional Culture, Pluripotent Stem Cells

## Abstract

Three-dimensional (3D) myocardial tissues for studying human heart biology, physiology and pharmacology have recently received lots of attention. Organoids as 3D mini-organs are created from multiple cell types (i.e. induced pluripotent stem cells (iPSCs) or embryonic stem cells (ESCs)) with other supporting co-cultured cells such as endothelial cells or fibroblasts. Cardiac organoid culture technologies are bringing about significant advances in organ research and allows for the establishment of tissue regeneration and disease modeling. The present review provides an overview of the recent advances in human cardiac organoid platforms in disease biology and for cardiovascular regenerative medicine.

## Introduction

Cardiovascular disease (CVD), which is a major cause of death globally, remains a great challenge for clinical remedy. CVD contains a wide array of disorders, including acute coronary syndrome, congenital heart disease, cerebrovascular disease, hypertension, arrhythmias, etc.^1^

The design and development of novel drugs and their application in disease treatments in the human model have many limitations. Also, the safety and efficacy of preclinical findings in two-dimensional (2D) cell cultures and animal models fail to represent human physiological conditions. To overcome these obstacles, three-dimensional (3D) cell models mimic human tissue-like, cell-cell, and cell-matrix interactions and enhance biological understanding during drug discovery and disease modeling.^2,3^

Organoids are 3D *in vitro* cell constructs that include multiple cell types (i.e. induced pluripotent stem cells (iPSCs) or embryonic stem cells (ESCs) with other supporting co-cultured cells such as endothelial cells or fibroblasts).^4^ By using the inherent self-assembly property of stem cells, organoids can be considered organ-like multicellular constructs with key characteristics including; 1) similarity to an original organ due to having more than one type of cell, 2) the existence of some specific functions related to the original organs including metabolic and physiologic function^5^, 3) having a resemblance to the native organ in terms of microscale tissue architecture, genes and protein expression.^6^

**Figure 1:**
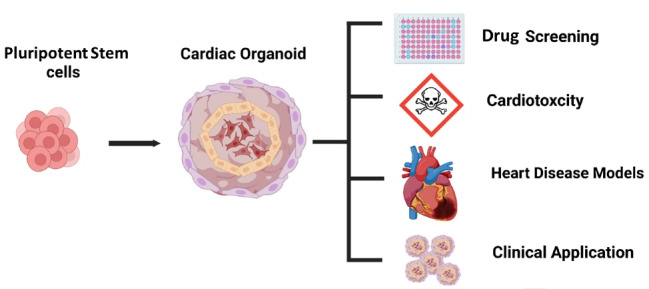
Human heart organoid potential applications

In recent years, the creation of the human heart organoid has received much attention due to its potential applications (Figure 1). In this review, we present an overview of the recent advances in heart organoid generation as novel tools for modeling human cardiac biology and disease.

## Materials and Methods

A literature search was done in electronic databases including PubMed, Cochrane Library, Scopus, and Google Scholar. A combination of the terms “organoid”, “cardiac organoid”, “heart organoid”, “stem cells”, “cardiac drug screening”, “regenerative medicine”, “three-dimensional cell culture”, “3D cell culture” were used. Case reports, editorial letters, gray literatures, unpublished reports and papers for which full texts were not available in English were considered as exclusion criteria. The latest date of these searches was on September 1, 2022. We reviewed recent knowledge about human cardiac organoid generation and summarized evidences in the realm of the generation of cardiac organoids, architectural design, cardiac organoids modeling in human cardiac diseases- *in vitro* and stem-cell based cardiac regeneration: limitations and gaps.

## Generation of cardiac organoids

Organoids are created from single cells/stem cells. Among the stem cells, either embryonic stem cells or tissue-resident, can be employed for this cultivation with special structural pillars and defined growth factors.^7^ After co-cultivation, the cells undergo self-organization and differentiation similar to the processes *in vivo*.^8^

The human heart consists of cardiomyocytes (~60%), with the remaining ~ 14% endothelial cells and approximately 24% cardiac fibroblasts as the principal non-myocyte cell type. The rest are a small number of smooth muscle cells, epicardial cells, conductance cells, and immune cells.^4,9^ The limited regenerative capacity of cardiomyocytes (CMs) in adult cardiovascular tissue is the main cause of its inability to repair itself or self-renew after injury.^10^

The advances in human induced pluripotent stem cells (hiPSCs) technology and cardiogenic differentiation resulted into an unlimited source of human cardiomyocytes for biomedical research. However, hiPSC-derived cardiomyocytes (hPSC-CMs) in traditional 2D culture lack many essential anatomical and physiological features, which hampers their capacity to predict human biology. A potential solution for this problem is multicellular 3D cardiac organoids.^11,12^

The development of 3D cardiac organoids culture conditions is faced with a significant challenge such as the identification of the precise primary and supporting cells ratio, culture media component, and specific conditions that mimic the biology of the tissue model. CMs differentiation of pluripotent stem cells (PSCs) with molecular, biochemical, and functional properties of adult CM is a very important challenge in organoid research techniques. In pioneering studies, the potential of human embryonic stem cell (hESC) - derived cardiac progenitor cells (CPC) for the development of mature CMs has been widely considered.^13,14^

## Architectural design

Structural pillars including collagen, matrigel or fibrin are another important prerequisite for the generation of cardiac organoids. Having biomimetic, perfusable vasculature, electromechanical integrity and conductivity properties are basic requirements for designing 3D cardiovascular tissue constructs.^15^

Although matrigel is the most commonly used matrix for 3D cultures of almost all kinds of epithelial and endothelial cells due to its close resemblance to the native extracellular matrix (ECM), using it has some disadvantages in organoid technology due to:^7,16^

- Complex composition
- Batch to Batch variation
- Lack of reproducibility in cell culture experiment
- Impossibility of being easily tailored to obtain specific organoid niches for specific organs

Given the undesirable properties of matrigel, other chemically and mechanically well-defined natural and synthetic scaffolds for organoid cultures have been evaluated recently. Considering their similarity to human ECM, some natural polymer-based hydrogels such as natural hydrogels, hyaluronic acid (HA), alginate, chitosan, PEG (Poly Ethylene Glycol)^17^ are preferred.

In general, Matrigel-based organoids lack the aforementioned essential properties, are not suitable for *in vivo* transplantation and cell therapy studies, and as a result, some natural alternatives to matrigel are preferred regarding their inherent bioactivity and similarity to human ECM.

## Cardiac organoids modeling in human cardiac diseases- *in vitro*

Organoids, which are morphologically and physiologically closest to organs with tissue-like *in vitro* conditions, offer a different and unique opportunity to study diseases. This issue becomes more important in the case of heart disease due to the special conditions of the heart. A group of cardiac diseases is defined as non-genetic diseases. Many Factors such as aging, interaction with other organs, diet, and toxicity can play an important role in causing these diseases. Myocardial infarction is defined as one of the most important and fatal diseases in this category. This disease begins with the interruption or severe restriction of blood flow in an area of the heart, causing hypoxia and subsequent scar tissue formation in this region, and the loss of cardiomyocytes in this way causes the heart to be unable to perform its function naturally. This situation leads to death or severely affects the patient’s quality of life. 3D organoid technology gives us this opportunity to model the developmental stages of this disease in vitro, to carefully examine the progression and course of this disease, and to obtain new results. Additionally, the survival of the surrounding cells can be able to regenerate damaged regions. These regeneration stages can also be investigated and researched by using organoids.

Genetic diseases are another category of heart disease. Genetic mutations in cardiomyocytes over time cause abnormal heart function, heart failure, or disease. In the field of these diseases, the use of organoids and disease modeling can also improve our knowledge about the process of development and treatment of these diseases. In 2016, Cashman et al were able to develop the first 3D human-engineered cardiac tissue model by using hiPSCs.^18^

In 2020, Lewis-Israeli et al presented a new method for producing human heart organoids using pluripotent stem cells. Human heart organoids (hHOs) were generated using a two-step conventional Wnt signaling modulation strategy under well-defined culture conditions. Further, this group was able to generate the first laboratory model of diabetes during pregnancy to study embryonic congenital heart defects.^19^ In 2019, Keung et al created human cardiac ventricular-like organoid chambers from pluripotent stem cells and investigated the effects of many drugs on these organoids.^20^ In 2018, Anderson et al generated a new heart organoid for the study of heart field/chamber-specific diseases.^21^

In a 2019 study, Zhao et al designed a cardiac tissue culture platform that was independent of cell source, and enabled drug testing under electrical pacing. They designed electrophysiologically different atrial and ventricular tissues with drug responses and different gene expression levels. This group managed to generate heteropolar cardiac tissues containing separate atrial and ventricular terminals for the first time. In this way, this group was able to model left ventricular hypertrophy with 8-month electrical conditioning.^22^

Recently, there has been a lot of research on organoid modeling of heart disease. The generation of these models will give us information about the formation and progression of different diseases and even their treatments.^23^

## Cardiac organoids for cardiovascular regenerative medicine

In human adults, the myocardium is one of the tissues that have a very low regeneration capacity. This feature of the myocardium causes heart failure conditions. Today, heart failure is the group of diseases that are the deadliest in the world. Recently, many studies by scientists have focused on triggering the endogenous regeneration of the heart. A group of studies is investigating why the regeneration of the heart tissue of mammals is active in the neonatal period. The discovery of this internal mechanism may be the hope for the adult human heart to regain the regeneration feature with the endogenous mechanisms.^24^

On the other hand, many scientists in the field of human regenerative medicine are investigating the use of pluripotent cells in the regeneration of heart tissue. One group of these studies focuses on the transplantation of cardiomyocytes differentiated from these cells into the heart tissue, while the other part is working on new factors that promote the regeneration of cardiac tissue.^25,26^ In 2017, Tiburcy et al were able to create a model of heart failure and heart repair using iPSCs and embryonic stem cells.^27^

Mills et al in a study conducted in 2017, examined the CM cell cycle arrest mechanism in detail in the human cardiac organoid that they created. The advantage of this research was the creation of the 3D phenotype and the study of this mechanism. In addition, in this study, the positive and negative effects of the components that play a role in cell proliferation, were investigated.^28^

In a study conducted by the same research group in 2019, the effect of 105 different small molecules with pro-regenerative potentials was investigated after cardiac organoids were created from hiPSCs.^29^

 In 2017, Voges et al investigated the capacity of regenerating human immature heart tissue using cardiac organoids. In this study, the potential of repairing the damaged tissue caused by dry ice, was evaluated for two weeks. The obtained results showed that immature human hearts can regenerate intrinsically.

It is very promising that organoids can create models of cardiac diseases and the results can used in the treatment of heart diseases, as well as researching regeneration mechanisms on 3D cardiac organoids and in human heart regeneration. These researches have been increasing recently and valuable information on heart regeneration will be obtained in the field of regenerative medicine.^30^

## Stem-cell based cardiac regeneration: Limitations and gaps

As shown in pioneering investigations, iPSCs-derived cardiomyocytes have the ability to contract, special potency for expressing the essential receptors for hormonal regulation of the myocardium and even forming gap junctions. Despite what was mentioned, prior to the initiation of cell based human clinical trials, more detailed investigations are needed in the realm of the risk of pluripotency-associated teratoma induced immunogenicity.^31-33^ Additionally, the long-term beneficial effects of a pure cell-based strategy (e.g. with iPSC) have a lot of flaws in terms of surviving in the injured myocardium micro-environment.^34^ Furthermore, based on recent evidences, organoids derived from ESCs may be rejected by the host immune system. In addition, since ESCs are created from early human embryos, they can be ethically problematic.^35^ Generally, inconsistencies in the reported engraftment rate, probability of tumorigenesis and the risk of arrhythmia may be the main concern of pluripotent stem cell-based therapies in CVDs.^36,37^

## Conclusion

Nowadays, human cardiac organoids for CVDs modelling have brought about considerable progress in the realm of maturation, improving cell therapy, and drug screening studies. This technology rapidly expands our knowledge in CVDs therapy. Given the improvement of organoid production technology, significant progress is expected in its application. At present, only one clinical trial using ESC- derived cardiomyocytes is ongoing.^38^ However, according to the mentioned limitations, more investigations in large- animal models are warranted to assess the safety and efficacy of cardiac regenerative therapy prior to translating these therapies to clinical trials.

## Acknowledgements

 The authors would like to thank the Cardiovascular Research Center of Tabriz University of Medical Sciences.

## Competing Interests

 The authors declare no conflict of interest.

## Funding

 This research project was funded by the Research Vice-Chancellor, Tabriz University of Medical Sciences under the grant (young assistant professors grant, 63669).
